# Cannabis use is associated with a lower likelihood of presence of HIV drug resistance mutations in a retrospective cohort of adults with HIV

**DOI:** 10.1515/nipt-2024-0010

**Published:** 2025-02-10

**Authors:** Jonathan F. Hale, Shellynea Reynolds, Heather R. Kates, Roberto D. Palella, Mohammed M. Benmassaoud, Kelly A. Smith, Daohai Yu, Servio H. Ramirez, Allison M. Andrews

**Affiliations:** Department of Pathology & Laboratory Medicine, Lewis Katz School of Medicine at Temple University, Philadelphia, PA, USA; Department of Pathology, Immunology and Laboratory Medicine, University of Florida College of Medicine, Gainesville, FL, USA; College of Public Health, Temple University, Philadelphia, PA, USA; Center for Biostatistics and Epidemiology, Lewis Katz School of Medicine at Temple University, Philadelphia, PA, USA; Department of Cancer and Cellular Biology, Lewis Katz School of Medicine at Temple University, Philadelphia, PA, USA; Center for Substance of Abuse Research, Lewis Katz School of Medicine at Temple University, Philadelphia, PA, USA

**Keywords:** HIV, HIV drug resistance mutations, substance use, cannabis, HIV positive clinical sample, odds ratios

## Abstract

**Objectives:**

A significant clinical concern in the era of Pre-Exposure Prophylaxis (PrEP) is the increased incidence of HIV Anti-Retroviral Drug Resistance Mutations (ARV-DRM). Previous research has indicated that there is an association between substance use and failed viral suppression, which can lead to ARV-DRM. The goal of this retrospective study was to investigate whether substance use as determined by at least one positive urinalysis screen is associated with increased/decreased odds of having a ARV-DRM.

**Methods:**

This study used firth logistic regression analyses of data retrieved from the National NeuroAIDS Tissue Consortium Data Coordinating Center to examine the relationship between substance use and ARV-DRM. The dataset analyzed 614 participants with the following criteria: HIV+ status, at least one paired plasma and cerebrospinal fluid (CSF) viral load measurement, at least one urinalysis of substance use, at least 18 years of age, and analysis of DRM in CSF/Plasma.

**Results:**

Cannabis use was a significant predictor of ARV-DRM and was associated with a lower odds of having ARV-DRM (odds ratio=0.189), after accounting for demographic variables and the interaction between polysubstance use and cannabis use. A significant negative relationship was observed between a cannabis positive test and high viremia (>1,000 copies/mL) but not between a cannabis positive test and CSF Escape (viral load CSF>viral load plasma).

**Conclusions:**

The above results may suggest an immunomodulatory role for cannabis that impacts the propensity for ARV-DRM. These findings could incentivize future research to further investigate effects of cannabis use on the development of HIV ARV-DRM.

## Introduction

Substantial strides have been made to turn HIV, a disease in which there is no vaccination or cure, into a chronic but manageable disease with the use of anti-retroviral therapy (ART) [1]. The goal of ART is to achieve viral suppression through significantly reducing viral replication, and thus, reduce the neurocognitive effects that accompany chronic HIV infection. One significant barrier to combating HIV is the emergence of anti-retroviral drug resistance mutations (ARV-DRM) within the HIV-infected population. HIV is particularly susceptible to replication issues inherent within the action of the virus’s reverse transcriptase, which are a source of ARV-DRM [2], 3]. Although these drug-resistant variants of HIV are less fit, selective drug pressure allows for these variants to continue to replicate within the infected individual and reduces the efficacy of ART-mediated viral suppression. One of the predominate issues with the development of ARV-DRM and the subsequent lack of viral suppression is cerebrospinal fluid (CSF) escape [4]. CSF escape occurs in 4–20 % of HIV-infected adults and is associated with an increase in neurocognitive symptomology and faster progression of the HIV infection, including HIV-associated dementia [4], 5]. CSF escape is diagnosed by meeting one of two criteria: either by having higher HIV RNA levels in the CSF than within the plasma, or by having differences in quantity or type of ARV-DRM in the CSF HIV RNA compared to plasma HIV RNA [4], 6], 7]. Not only does this pose a risk for individuals already infected with HIV, but also allows for transmitted drug resistance (TDR) to another individual.

ART has been adjusted to be used as a prophylaxis for individuals who are currently HIV-negative but at high risk for HIV infection. Pre-exposure Prophylaxis (PrEP) prevents viral replication and thus prevents the establishment of a chronic infection [8]. The efficacy of PrEP has been reported in a number of trials and although highly dependent on the level of medication adherence, has shown significant reduction in HIV infections among high risk individuals [9]. In humans, there have only been six reported cases of HIV infection in individuals who were taking PrEP and adhering to the medication regimen [10]. Of those six cases, five of them were infected with an HIV strain that had ARV-DRM. Although the number of individuals utilizing PrEP is growing, the presence of ARV-DRM within the HIV-infected population undermines these efforts [11], 12]. While PrEP has been an effective prophylaxis treatment, there has been a growing interest in research to understand the development of ARV-DRM in the PrEP era and whether there are specific precipitating factors that increase the incidence of ARV-DRM.

Previous research has identified that drugs of use, misuse and addiction affect every aspect of HIV, including viral replication, transmission, pathogenesis, treatment efficacy and adherence [13], 14]. Stimulants (such as cocaine, crack cocaine, methamphetamine, etc.) have a well-documented association with adverse HIV outcomes (e.g. low CD4 counts, viral load) both in clinical and basic science research [15], [16], [17], [18], [19], [20]. Drug users are more likely to experience gaps in treatment adherence [21], 22] and drugs of use, misuse and addiction may impact ART metabolism and distribution in the brain [23], [24], [25]. However, most studies on the effect of addictive substances on viral suppression do not specifically examine whether this reduced viral suppression places an HIV positive individual at an increased risk for ARV-DRM. The current study sought to investigate whether there was a relationship between a history of substance use and the likelihood of having HIV ARV-DRM among a clinical sample of HIV positive individuals. Previous findings have suggested that substance use may be a mechanism that leads to drug resistance mutations among the HIV positive population. Thus, we hypothesized that there will be a significant and positive relationship between HIV ARV-DRM and substance use in this study cohort.

## Materials and methods

### Participants

We obtained data through the National NeuroAIDS Tissue Consortium (NNTC) Data Coordinating Center, which included data from the NNTC and Central Nervous System HIV Anti-Retroviral Therapy Effects Research (CHARTER) between January 2005 and September 2016. The criteria for participant inclusion included the following eligibility criteria for our analysis: HIV+ status, at least one paired plasma and cerebrospinal fluid viral load measurement, at least one urinalysis of substance use, at least 18 years of age, and analysis of DRM in CSF/Plasma (Table S1). The DRM analysis has been detailed in previous studies [4], [26], [27], [28].

### Outcomes of interest

The outcome of interest for the main analyses presented here was the presence of an ARV-DRM, which was scored as positive if participants had a mutation in either the cerebrospinal fluid or plasma for either protease inhibitors (PI) or nucleoside reverse transcriptase inhibitors/non-nucleoside reverse transcriptase inhibitors (NRTI/NNRTI) as determined by lab tested plasma and cerebrospinal fluid for HIV ARV-DRM and viral levels.

### Predictors of interest

#### Substance use

The substances included in our analyses were: cannabis, amphetamine, cocaine, sedatives, opiates. Substance use status was determined by urinalysis data that were collected multiple times during the study participant’s enrollment. Urinalysis results were summarized and analyzed per-participant rather than per-visit to reduce potential bias due to inconsistent numbers of visits across participants: if a participant screened positive for a substance one or more times, they were scored positive for that substance. Some substance categories in the urinalysis were combined due to the similarity of the substances (e.g. amphetamines and methamphetamine; opioids, oxycodone, buprenorphine, and methadone) with respect to their actions on the central nervous system [29]. Participants that screened positive for two or more substances were scored as positive for polysubstance use. Note, our analyses did not include tobacco or alcohol use which was not analyzed in the urinalysis.

### Other variables considered

We captured and considered the relationship between non-substance use factors on substance use or on the outcome variable. These included sociodemographic characteristics (age at survey enrollment, race/ethnicity, and sex) and study-related characteristics (length of infection). We conducted post-hoc analyses to test two additional outcome variables, CSF escape (viral load CSF>viral load plasma), and high viremia (>1,000 copies/mL), based on the observed relationship between cannabis use and our primary outcome variable. CSF escape and high viremia which were scored and analyzed for each study participant per-visit based on laboratory measurements of HIV-1 RNA levels measured in cell-free CSF and plasma (Table S3).

### Statistical analyses

We used Firth logistic regression analyses to estimate the odds ratios (ORs) and determine the significance of associations between substance use and ARV-DRM status. Firth regression was selected to address the small number of ARV-DRM cases in certain subgroups. First, we fitted univariable models for each predictor of interest (substance) and each additional variable under consideration. We then fitted multivariable models that included combinations of predictors significant at the p<0.20 level in univariable analyses (Table S2) (General “Substance use” was excluded from multivariable models because of its association with specific substance use is perfectly confounded). The best-fitting multivariable model that included cannabis (as determined by lowest LRT p-value) included Cannabis, Sex, and Race. The best-fitting multivariable model that included IV drug use included IV Drug Use, Length of Infection, and Race.

We accounted for the significant correlation between cannabis and polysubstance use (Pearson’s Chi-squared test) through additional analyses: (1) fitting a model with polysubstance use as a covariate; (2) fitting a model with an interaction between cannabis and polysubstance use to test if the ARV-DRM and cannabis relationship varied by polysubstance use; and (3) stratifying by polysubstance use and fitting the original model (Cannabis, Sex, Race) within each subgroup. Additionally, to address the influence of the rare class (n=1) of cannabis-positive, polysubstance-negative, ARV-DRM-positive participants on the significant result, we conducted a sensitivity analysis by simulating 43 new participants proportionally based on the existing dataset’s distributions of cannabis use, polysubstance use, sex, and race (Table S4). (This was the number of simulated participants needed to add a single case to the rare class.) These participants were added to the dataset, and the updated data were analyzed using Firth’s logistic regression to assess the robustness of the original model’s (Cannabis × Polysubstance + Sex + Race) results. (No additional testing was done for IV drug use, as its correlation with polysubstance use was not significant.)

To explore possible mechanisms for the significant relationship between cannabis use and ARV-DRM in our dataset, we tested the relationships between Cannabis use and CSF escape and between Cannabis use and high viremia. These tests were performed using the participant by visits dataset (n=2,343) (Table S3) and a generalized linear mixed model (GLMM) with a binomial distribution to assess the association between Cannabis use and outcome variable, including participant ID as a random effect to account for repeated measures. Unlike ARV-DRM which is scored once per participant and analyzed using the summarized by-participant dataset (Table S1), CSF escape and high-viremia may have different measurements at different times and be directly associated with proximal substance use as recorded by per-visit urinalysis. To isolate the effect of Cannabis from other substances, we did not consider visits where a participant tested positive for substances other than Cannabis.

All analyses were performed using R [30] Firth Logistic regression models were fitted using the logistf() function from the logistf package in R [31]. Person’s Chi-square test was performed with the chisq.test() function in R. Generalized linear mixed model were fitted using the glmer() function from the lme4 package in R [32] Odds ratios (OR) were calculated by exponentiating the model coefficients, and 95 % confidence intervals (CI) for the ORs were derived using the confint() function in R. Data were simulated using the sample() function in R with replacement and proportions from the original dataset. Predictors with p-values <0.05 were considered statistically significant.

## Results

### Sample characteristics

A total of 614 participants met eligibility criteria for the current study and are described in Table 1. The sample consisted of 80.75 % men, 45.25 % White, 37.36 % Black, 9.66 % Multi-racial, 6.19 % Hispanic, and ≲1 % Unknown, Asian, or Native American. Age ranged from 18 to 72 at time of enrollment in the study (M=45.15, SD=8.57). Cannabis was the most frequent urinalysis result, with positive screenings in 60.84 % of participants, followed by opiates (28.50 %), cocaine (28.02 %), no substances (21.10 %), sedatives (15.97 %), and amphetamines (12.90 %). Additionally, 46.74 % of participants tested positive for polysubstance use. Of the polysubstance use participants, 82.58 % screened positive for cannabis.

**Table 1:** Selected characteristics and substance use characteristics of participants with and without ARV-DRM age 18 and older with HIV+ status, at least one paired plasma and cerebrospinal fluid viral load measurement, at least one urinalysis of substance use (n=618). Data from the NNTC and Central Nervous System HIV Anti-Retroviral Therapy Effects Research (CHARTER) between January 2005 and September 2016. p-values are reported for the univariable test of the effect of the variable on ARV-DRM. IV drug use status was available for a subset of participants indicated by the secondary heading.

Characteristic	Total sample n=618		ARV-DRM present (n=20)		ARV-DRM absent (n=598)		p-Value
	n	(%)	n	(%)	n	(%)	
Sex							
Female	117	19.06	0	0	117	19.70	NA (ref)
Male	497	80.94	20	100	477	80.30	0.02
Race/ethnicity							
White	275	44.79	10	50	265	44.61	NA (ref)
Black	232	37.79	8	40	224	37.71	0.92
Hispanic	0	0.00	0	0	0	0.00	0.99
Multi-racial	59	9.61	0	0	59	9.93	0.17
Unknown	46	7.49	2	10	44	7.41	0.63
Asian	1	0.16	0	0	1	0.17	0.29
Native American	1	0.16	0	0	1	0.17	0.29
Substance use	483	78.66	10	50	473	79.10	NA (ref)
No substances	131	21.34	10	50	121	20.37	0.003
Cannabis	375	61.07	9	45	366	61.62	0.13
With polysubstance	237	38.60	8	40	229	38.55	0.82
Without polysubstance	138	22.48	1	5	137	23.06	0.02
Amphetamines	80	13.03	4	20	76	12.79	0.28
Cocaine	174	28.34	4	20	170	28.62	0.45
Sedatives	99	16.12	2	10	97	16.33	0.56
Opiates	177	28.83	6	30	171	28.79	0.83
Polysubstance	287	46.74	9	45	278	46.80	0.88

Characteristic	Total sample n=218		ARV-DRM present (n=3)		ARV-DRM absent (n=215)		p-Value
	n	(%)	n	(%)	n	(%)	
IV drug use	60	27.52	2	66.66	1	33.33	0.14

All twenty participants that had an ARV-DRM had a mutation in both the cerebrospinal fluid and plasma for both protease inhibitors (PI) and nucleoside reverse transcriptase inhibitors/non-nucleoside reverse transcriptase inhibitors (NRTI/NNRTI). The participants with ARV-DRM consisted of 100 % men, 50 % white, 40 % black, and ranged in age from 24 to 62 at time of enrollment in the study (M=45.85, SD=11.15). No substance use was the most frequent urinalysis result among this sub-sample, accounting for 50 % of ARV-DRM positive participants. Of the forty-five percent of ARV-DRM positive participants who tested positive for cannabis, most (88.89 %) screened positive for other substances as well.

CSF escape was also characterized in study participants with HIV ARV-DRM (Table S1). Among the 20 participants with ARV-DRM, 13 had greater viral loads (VL) in their CSF compared to their plasma at least once during the study (Mean Percent of Visits with CSF escape=20.76 %, SD=0.16). Half the participants with an ARV-DRM had discord in the mutations found in the CSF/plasma).

### ARV-DRM and substance use

In a Firth logistic regression analysis of the relationship between Cannabis use and ARV-DRM that included covariates for Sex, Race, and the interaction between Cannabis use and Polysubstance use, participants who used cannabis had lower odds of having ARV-DRM than participants who did not use cannabis (OR=0.18, 95 % CI=0.02, 0.8, p-value=0.022) (Table 3). Cannabis use was only marginally significant (OR=0.46, 95 % CI=0.19, 01.13, p-value=0.089) in the model fit without Polysubstance use (Table 2), and there was a highly significant correlation between Cannabis use and Polysubstance use (p-value<2.2e-16). Polysubstance use was not a significant predictor in the multivariable or univariable model.

**Table 2:** Results of adjusted Firth logistic regression analyses for the association between HIV ARV-DRM and substance use. All predictors with p<0.20 in univariable analyses were included in the multivariable models tested. Data from the NNTC and Central Nervous System HIV Anti-Retroviral Therapy Effects Research (CHARTER) between January 2005 and September 2016.

Predictors	Sample	p-Value	Odds ratio	95 % CI (OR)
Cannabis + Sex + Race	Full sample (n=618)	0.0843	0.46	0.18, 1.11
Length of Infection + Race + IV Drug Use	IV use scored (n=218)	0.0994	5.70	0.71, 65.76

IV drug use had a p-value of <0.2 in a univariable model of the effect of IV drug use on ARV-DRM (Table 1), but IV drug use was not significant in this or in the best-fitting multivariable model that included IV drug use (OR=5.7, 95 % CI=0.71, 65.76, p-value=0.099) (Table 2). No other substance use (Amphetamine, Cocaine, Sedatives, Opiates) was a significant predictor of ARV-DRM (Table 1).

### ARV-DRM, CSF escape, high-viremia, and cannabis use

Because of the complex relationship between cannabis use and ARV-DRM in our dataset, we performed additional analyses (Table 3). Our sensitivity analysis demonstrated when an additional ARV-DRM positive and cannabis positive user was added to the non-poly substance user dataset via an addition of simulated participants, the p-value of cannabis was only marginally significant (OR=0.30, 95 % CI=0.06, 1.04, p-value=0.059). We did not find a significant relationship between Cannabis use and CSF-escape, however we did find that Cannabis use is associated with a decreased odds of high viremia (OR=0.66, p-value=0.04).

**Table 3:** Results of additional adjusted analyses for the relationship between HIV ARV-DRM, CSF-escape, high-viremia, and cannabis use. Polysubstance use was included in models or used to stratify the data due to its significant correlation with cannabis use. Data from the NNTC and Central Nervous System HIV Anti-Retroviral Therapy Effects Research (CHARTER) between January 2005 and September 2016.

Predictors	Sample	p-Value	Odds ratio	95 % CI (OR)
Outcome variable: **ARV-DRM**				
Cannabis + Polysubstance + Sex + Race	Full sample (n=618)	0.0631	0.38	0.13, 1.05
Cannabis × Polysubstance + Sex + Race	Full sample (n=618)	0.0173	0.17	0.02, 0.76
Cannabis + Sex + Race	No polysubstance (n=327)	0.0251	0.2	0.02, 0.83
Cannabis + Sex + Race	Polysubstance (n=287)	0.9741	1.03	0.21, 10.51
Cannabis × Polysubstance + Sex + Race	Sensitivity test (n=657)	0.0589	0.30	0.06, 1.04
Outcome variable: **CSF-escape**				
Cannabis + (1|Participant ID)	All visits (no polysubstance users) (n=2,343)	0.2190	1.34	0.84, 2.13
Outcome variable: **High viremia**				
Cannabis + (1|Participant ID)	All visits (no polysubstance users) (n=2,343)	0.0396	0.66	0.45, 0.98

## Discussion

In this retrospective study of HIV positive participants, cannabis use was significantly associated with reduced odds of having ARV-DRM in a subsample of participants that did not test positive for polysubstance use. This relationship is also supported by a significant result in a model that includes the full sample and adjusts for the interaction between Cannabis and polysubstance use and is potentially related to our finding that Cannabis use is associated with lower odds of high-viremia. Taken together, this provides evidence for a negative relationship between cannabis use and presence of ARV-DRM, and no significant positive relationships between substance use and presence of ARV-DRM.

The significance of cannabis use in relation to ARV-DRM only emerges when the interaction between cannabis and polysubstance use is included in the analysis, despite the interaction term itself not being statistically significant. This pattern suggests a lack of stability in these relationships, likely due to the small sample size, the complexity and overlap of substance use behaviors, and the rarity of ARV-DRM [33]. A larger sample size may help clarify these associations and provide more robust evidence. A sensitivity analysis demonstrated that when the rare class of polysubstance use with cannabis and ARV-DRM was increased from n=1 to n=2 through simulating a representative additional sample of 42 participants, Cannabis use was marginally significant (p=0.06, OR=0.3).

Of particular interest in this study were the results of Cannabis being a potentially protective factor for the development of an ARV-DRM. Cannabis is widely recognized for its actions on the cannabinoid type 1 and 2 (CB1 and CB2) receptors. CB1 is typically considered psychotropic while CB2 receptors are considered the anti-inflammatory cannabinoid receptor [34]. Several studies have found that CB2 agonists or THC reduces blood-brain barrier dysfunction, activation of macrophages, and HIV/SIV replication [35], [36], [37]. Our analysis found a significant negative relationship between cannabis and high viral load. Thus, a possible explanation for the reduced likelihood of HIV ARV-DRM in cannabis using participants could be that with reduced neuroinflammation and attenuation of viral replication, there is a reduction in the accumulation of viral quasispecies that acquire an advantageous drug resistance mutation. This notion is supported by a recent study that found reduced odds of tissue harboring HIV-1 DNA and viral DNA copies in brain and peripheral tissue in people using cannabis [38]. In that study, inflammatory cytokine levels were also significantly lower in lymphoid tissues [38]. Others studies have also found reduced inflammatory markers in the CSF and blood in recent cannabis users [39].

We also performed an analysis to determine whether there were relationships between drug use, CSF escape and ARV-DRM. No significant relationships were found between cannabis and CSF escape, or between ARV-DRM and CSF escape. In light of these results, future research should further investigate the effects of cannabis on the development of HIV ARV-DRM and on CSF escape.

Although there is some evidence of the potential benefits of cannabis use with HIV infection, there are also studies that do not support this conclusion. Roth [40] and Thames [41] have shown that cannabis over-suppresses the immune system which can allow for increased viral replication and worsened neurocognitive symptomology [40], 41]. In addition to these studies on potential negative effects of cannabis, several studies have found no differences in the effect of cannabis in viral load [42], 43] in contrast to our cohort.

Based on prior studies [44], [45], [46], [47], [48], [49] we expected to see an increased risk for ARV-DRM among stimulant using participants and intravenous substance using participants. One of the reasons this study may not have found similar results could be the small percentage of participants who endorsed solely stimulant use and intravenous substance use. Moreover, the participants that used stimulants and used substance intravenously also used cannabis, which could have overshadowed the harmful effects of these behaviors. Although we did not find support for increased ARV-DRM in stimulant or intravenous substance users within our study, the positive significant relationship between amphetamines and CSF escape suggests that these relationships may be complex and worth further study.

The current study has several limitations that should be addressed. First, the proportion of the sample that had HIV strains with ARV-DRM was very low, only accounting for slightly over 3 % of the total sample. Due to this low incidence, it is difficult to make substantial claims about the results our data shows. We performed an analysis of data from a prior study [4] that is comprised of a cohort of participants from the CHARTER-HNRC and CHARTER-NNTC cohorts. ART adherence, which can contribute to the development of ARV-DRMs, was not evaluated. Drug users have been reported to have reduced ART adherence [50]. However, several studies note that cannabis users did not have issues with ART adherence [22]. However, one study found that individuals with cannabis dependence [22] and older individuals has suboptimal ART adherence [21]. Our analysis which indicated that cannabis users had reduced risk of ARV-DRMs does not suggest that these individuals had suboptimal ART adherence. If these cannabis users did have suboptimal ART adherence, it may suggest a larger effect than we have measured. The prior analysis of the cohort [4] found that PI regimens increased the risk for mutations. While we could not perform an analysis to examine whether individuals on PIs were more or less likely to have used cannabis, we do not feel that cannabis use would impact the probability of an individual to be on a PI regimen.

With the rise of the use of PrEP, there is a need to monitor changes in HIV quasispecies in order to prevent spread through resistance mutations. However, it’s important to note that new PrEP formulations are on the horizon and some regimens are more resilient against ARV-DRMs. Consequently, the risk of transmission of HIV with ARV-DRMs may decrease as therapeutics evolve.

Future research should have a sample with a larger proportion of HIV ARV-DRM or equal representation of ARV-DRM patients and non-ARV-DRM patients. Additionally, the way in which substance use was analyzed has notable limitations. Within the original data collection there was an inconsistent number of participants visits for urinalysis across the sample. To account for this, a participant was coded as a substance user with a single positive urinalysis screening. Subsequent studies with a higher frequency of ARV-DRM participants should use ranked analyses under a consistent number of urinalysis screenings or use a psychiatric assessment of substance use disorders. Additionally, drug use was evaluated based on urinalysis data which does not track prior medical diagnoses (substance use disorders, dependence etc.) or self-reported drug use.

In summary, this study provides preliminary data for the association between substance use and HIV ARV-DRM. Specifically, this research found that cannabis use is significantly associated with a decreased likelihood of HIV ARV-DRM. More research should focus on the potential protective and risk factors of addictive substances in clinical and *in vivo* settings to identify the potential mechanism of action that these substances are having on HIV pathology, replication, and neurocognitive symptomology. Finally, in the era of PrEP, it is becoming urgently necessary to understand any factors that may affect the development of ARV-DRM and the spread of these quasispecies through the population.

## Conclusions

The presence of HIV transmitted drug resistance is of growing concern within the clinical community during the era of pre-exposure prophylaxis for individuals at high risk for HIV infection. Infection by an HIV quasispecies that is resistant to the standard prophylaxis medication renders the treatment ineffective and allows for the establishment of chronic HIV infection. To date, there is little published work that has investigated the association between drugs of use, misuse and addiction and the risk of development of anti-retroviral drug resistance mutations within a clinical sample of HIV positive individuals. We have found that cannabis use is significantly associated with a decreased likelihood of HIV ARV-DRM and suggest that cannabis use may be a protective factor against the development of anti-retroviral drug resistance mutations. More research is necessary to identify the mechanism in which addictive substances act on HIV viral suppression and on the development of anti-retroviral drug resistance mutations.

## Supplementary Material

Supplementary Material Details

Supplementary Material Details

Supplementary Material Details

Supplementary Material Details

Supplementary Material Details
