# Supplementary material for: Cannabis use is associated with a lower likelihood of presence of HIV drug resistance mutations in a retrospective cohort of adults with HIV
Source: NeuroImmune Pharm Ther. 2025 Feb 10;4(1):49–57. doi: 10.1515/nipt-2024-0010 (PMC12041849; doi:10.1515/nipt-2024-0010)
Supplement: Supplementary file 1 — Supplementary Material Details [file j_nipt-2024-0010_suppl_001.docx]

**Table S1. Dataset:** Urinalysis results, demographic characteristics, and ARV-DRM status summarized per-participant used for analyses in Tables 1, 2, and 3. CSF-escape characterization for ARV-DRM positive participants is also included and is described in the results but are not included in a statistical analysis.

**Table S2.** **Results** of Firth logistic regression models evaluating predictors of antiretroviral drug resistance mutation (ARV-DRM) status. Variables included in these models had p < 0.2 in a univariable model of ARV-DRM ~ Var (Table 1). P-values are reported for a likelihood ratio test (LRT) comparing the full model to the null model (intercept-only, containing no predictors). The LRT p-value indicates whether the full model significantly improves the fit compared to the null model. Significant predictors in each full model, along with their corresponding p-values, are reported. All predictor combinations were tested to identify the models that best explain ARV-DRM status. Best-fitting models are summarized in Tables 2 and 3.

**Table S3. Dataset:** Urinalysis results, CSF escape score, high-viremia score, and ARV-DRM status for all participants x visits. CSF escape is defined as cerebrospinal fluid viral load (CSF VL) greater than plasma viral load (Plasma VL), and high-viremia is scored as positive as viral load >1000 copies/mL. Visits with positive urinalysis results for substances other than Cannabis were excluded from the analyses.

**Table S4. Dataset:** Data for Cannabis use, sex, race, polysubstance use, and ARV-DRM status from Table S1 plus simulated values from 43 simulated participants used for analysis in Table 3 outcome variables CSF-escape and High-viremia.
